# Earlier onset of proteinuria or hypertension is a predictor of progression from gestational hypertension or gestational proteinuria to preeclampsia

**DOI:** 10.1038/s41598-021-92189-w

**Published:** 2021-06-16

**Authors:** Mamoru Morikawa, Michinori Mayama, Kiwamu Noshiro, Yoshihiro Saito, Kinuko Nakagawa-Akabane, Takeshi Umazume, Kentaro Chiba, Satoshi Kawaguchi, Hidemichi Watari

**Affiliations:** Department of Obstetrics and Gynecology, Hokkaido University Graduate School of Medicine, Kita-ku N15 W7, Sapporo, 060-8638 Japan

**Keywords:** Medical research, Risk factors

## Abstract

Although gestational hypertension (GH) is a well-known disorder, gestational proteinuria (GP) has been far less emphasized. According to international criteria, hypertensive disorders of pregnancy include GH but not GP. Previous studies have not revealed the predictors of progression from GP to preeclampsia or those of progression from GH to preeclampsia. We aimed to determine both sets of predictors. A retrospective cohort study was conducted with singleton pregnant women who delivered at 22 gestational weeks or later. Preeclampsia was divided into three types: new onset of hypertension/proteinuria at 20 gestational weeks or later and additional new onset of other symptoms at < 7 days or at ≥ 7 days later. Of 94 women with preeclampsia, 20 exhibited proteinuria before preeclampsia, 14 experienced hypertension before preeclampsia, and 60 exhibited simultaneous new onset of both hypertension and proteinuria before preeclampsia; the outcomes of all types were similar. Of 34 women with presumptive GP, 58.8% developed preeclampsia; this proportion was significantly higher than that of 89 women with presumptive GH who developed preeclampsia (15.7%). According to multivariate logistic regression models, earlier onset of hypertension/proteinuria (before or at 34.7/33.9 gestational weeks) was a predicator for progression from presumptive GH/GP to preeclampsia (odds ratios: 1.21/1.21, P value: 0.0044/0.0477, respectively).

## Introduction

According to the conventional criteria of hypertensive disorders of pregnancy, women with proteinuria alone (gestational proteinuria; GP) or hypertension alone (gestational hypertension; GH) are not diagnosed to be experiencing preeclampsia until they exhibit additional hypertension or proteinuria; those who do not develop hypertension or proteinuria are diagnosed with GP or GH, respectively, at 12 weeks postpartum^1^. Accordingly, GP or GH is the retrospective diagnosis. Without progression to preeclampsia until 12 weeks postpartum, GP or GH might be presumptive GP or presumptive GH^2^. Women with GP or GH are commonly diagnosed with preeclampsia with additional hypertension or proteinuria until 12 weeks postpartum (proteinuria preceding preeclampsia; P-PE, or hypertension preceding preeclampsia; H-PE).

Although GH is a well-known disorder, GP has not been emphasized and GH has received far less emphasis. In 2013, the American College of Obstetricians and Gynecologists revised its criteria of preeclampsia^3^, and in 2014, the International Society for the Study of Hypertension in Pregnancy revised its criteria of hypertensive disorders of pregnancy^4^. According to both sets of criteria, hypertensive disorders of pregnancy include GH but not GP. The Japanese criteria for diagnosing preeclampsia were revised in 2018 based on these revisions in the criteria of preeclampsia^5^.

Some previous studies have described the relationships between GH and preeclampsia. Women with GH and those with preeclampsia shared most risk factors: early gestational age at delivery, including overweight and obesity, primipara, history of preeclampsia, types 1 and 2 diabetes mellitus, and twin or higher-order multiple pregnancies^6^. GH is associated with a higher risk for maternal and neonatal morbidity compared with mild preeclampsia (hypertension plus proteinuria) without superimposed preeclampsia^7^. Furthermore, in the progression of GH to severe preeclampsia, the presence of proteinuria was associated with subsequent maternal and neonatal complications^8^.

In studies conducted since 2000, the severity of proteinuria during pregnancy has been considered to have limited clinical significance in terms of its effect on maternal and/or perinatal outcomes. Previous studies^9–12^ have demonstrated no association between severe proteinuria and poor perinatal outcomes among women with preeclampsia. Conversely, other studies^13–15^ have demonstrated that severe proteinuria was associated with poor perinatal outcomes among women with preeclampsia. Morikawa et al. found that the optimal protein/creatinine ratio cutoff values for determining early preterm birth and maternal central serous chorioretinopathy were 4.1 and 4.8, respectively, in women with preeclampsia^16^.

Women with new-onset GP in the absence of hypertension, particularly those with earlier onset, may exhibit a higher likelihood of progression to preeclampsia compared with women with a presumptive diagnosis of GH^2^. Therefore, the statement that “the outcome of pregnancy in patients with isolated proteinuria is favourable”^3,4^ is misleading. Physicians should be aware of the progression from presumptive GP to preeclampsia when counseling patients^17^.

In previous studies, the predictors of progression from GP to preeclampsia and from GH to preeclampsia were not elucidated. Therefore, the present study aimed to determine these predictors and to clarify the relation between GP, GH, and preeclampsia (hypertension plus preeclampsia) that develops from GP or GH.

## Results

### Frequencies of gestational proteinuria, gestational hypertension, and preeclampsia

In the present study, we enrolled 183 pregnant women (14 with GP, 75 with GH, and 94 with preeclampsia diagnosed as hypertension plus proteinuria). Among the 169 women with the hypertensive disorders of pregnancy (GH or preeclampsia), the frequency of preeclampsia was 55.6%. In the 94 women with preeclampsia, 20 (21.3%) exhibited P-PE, 14 (14.9%) experienced H-PE, and 60 (63.8%) had “Simultaneous preeclampsia” (S-PE) (Supplemental Fig. 1).

The number of women in whom GP progressed to P-PE (20 [58.8%] of 34) was significantly higher than that of women in whom presumptive GH progressed to H-PE (14 [15.7%] of 89; P < 0.0001).

### Characteristics and outcomes of the pregnant women in five groups

Table 1 lists the characteristics and outcomes of women with GP, P-PE, GH, H-PE, and S-PE. Age and body mass index before pregnancy were similar among the groups.

**Table 1:** Characteristics and outcomes of participants.

Characteristic	Presumptive gestational proteinuria		Presumptive gestational hypertension		E. Simultaneous proteinuria and hypertension preceding preeclampsia (n = 60)	P < 0.05
	A. Gestational proteinuria (n = 14)	B. Proteinuria preceding preeclampsia (n = 20)	C. Gestational hypertension (n = 75)	D. Hypertension preceding preeclampsia (n = 14)		
Age (years) ^a^	33.5 [24.3–37.5]	33.5 [29.0–37.5]	36.0 [33.0–38.0]	36.5 [32.3–39.0]	34.0 [30.3–38.0]	
Primipara (%)	8 (57.1%)	14 (70.0%)	46 (61.3%)	9 (64.3%)	43 (71.7%)	
Body mass index before pregnancy^a^	20.9 [18.7–37.5]	19.8 [18.5–22.1]	22.7 [20.1–25.8]	22.0 [20.9–25.4]	21.1 [19.4–23.6]	
**Gestational weeks**						
At the onset of hypertension^a^	–	34.4 [31.4–35.9]	36.9 [34.3–38.1]	32.9 [30.2–34.7]	35.4 [29.3–38.0]	C vs. D
At the onset of proteinuria^a^	35.7 [35.7–37.2]	31.5 [29.2–33.9]	–	35.3 [24.3–37.5]	35.9 [28.9–37.8]	A vs. B
At the diagnosis of preeclampsia^a^	–	34.4 [31.4–35.9]	–	35.3 [32.8–36.2]	35.9 [29.3–38.0]	
Early-onset preeclampsia (< 34 gestational weeks at diagnosis of preeclampsia; %)	**–**	9 (45.0%)	**–**	5 (35.7%)	24 (40.0%)	
At delivery^a^	37.6 [36.4–39.4]	34.9 [32.1–36.7]	37.7 [36.1–38.7]	36.5 [33.8–37.5]	37.0 [31.3–38.5]	B vs. A B vs. C
Preterm birth (< 37 gestational weeks at delivery; %)	4 (28.6%)	15 (75.0%)	21 (28.0%)	9 (64.3%)	29 (48.3%)	C vs. B C vs. D C vs. E
Early preterm birth (< 34 gestational weeks at delivery; %)	0 (0.0%)	8 (40.0%)	11 (14.7%)	4 (28.6%)	23 (38.3%)	A vs. B A vs. E C vs. E
Cesarean section (%)	5 (35.7%)	16 (80.0%)	44 (58.7%)	13 (92.9%)	46 (76.7%)	A vs. B A vs. D A vs. E C vs. D C vs. E
**Perinatal outcomes**						
HELLP syndrome (including partial type) (%)	0 (0.0%)	5 (25.0%)	1 (1.3%)	3 (21.4%)	11 (18.3%)	C vs. B C vs. D C vs. E
Abruptio placentae (%)	0 (0.0%)	1 (5.0%)	2 (3.3%)	0 (0.0%)	2 (3.3%)	
Eclampsia or posterior reversible encephalopathy syndrome (%)	1 (7.1%)	1 (5.0%)	0 (0.0%)	1 (7.1%)	3 (5.0%)	
Pulmonary edema (%)	0 (0.0%)	3 (15.0%)	0 (0.0%)	3 (21.4%)	9 (15.0%)	C vs. B C vs. D C vs. E
Peripartum cardiomyopathy (%)	0 (0.0%)	1 (5.0%)	0 (0.0%)	0 (0.0%)	2 (3.3%)	
Central serous chorioretinopathy (%)	0 (0.0%)	2 (10.0%)	0 (0.0%)	0 (0.0%)	2 (3.3%)	
Maternal death (%)	0 (0.0%)	0 (0.0%)	0 (0.0%)	0 (0.0%)	0 (0.0%)	
**Neonatal outcomes**						
Birth weight (g)^a^	2598 [2406–3053]	1873 [1269–2590]	2829 [2126–3160]	2130 [1451–2561]	2220 [1096–2753]	A vs. B C vs. B C vs. D C vs. E
Birth weight (standard deviation)^a,^^**b**^	0.02 [$-$ 1.63–0.63]	$-$ 1.01 [$-$ 2.27 ${-} -$ 0.20]	0.13 [$-$ 1.10–1.04]	$-$ 1.01 [$-$ 2.17 ${-} -$ 0.18]	$-$ 1.01 [$-$ 1.87 ${-} -$ 0.09]	C vs. B C vs. E
Light-for-date infant (%)	4 (28.6%)	8 (40.0%)	15 (20.0%)	4 (28.6%)	17 (28.3%)	
Apgar score < 8 at 5 min (%)	0 (0.0%)	2 (10.0%)	3 (4.0%)	1 (7.1%)	10 (16.7%)	
Stillbirth or early neonatal death (%)	0 (0.0%)	1 (5.0%)	2 (2.67%)	1 (7.1%)	5 (8.3%)	

^a^Data are listed as median (25th percentile–75th percentile).

^b^Birth weight (standard deviation) are calculated according to normative data for Japanese infants^33^.

*HELLP* hemolysis, elevated liver enzyme levels, and low platelet count.

Supplemental Fig. 2A lists gestational weeks (GWs) at the onset of GP or GH, onset of preeclampsia, and delivery. In this study, the onset of proteinuria in P-PE occurred significantly earlier compared with that in GP (P = 0.0276), and the onset of hypertension in H-PE occurred significantly earlier compared with that in GH (P = 0.0150). However, the onset of preeclampsia (hypertension plus proteinuria) occurred at similar GWs in women with P-PE, H-PE, and S-PE. Furthermore, women with P-PE, H-PE, and S-PE delivered at similar GWs. The frequency of preterm birth (< 37 GWs at delivery) was significantly lower among women with GH than among those with P-PE (P = 0.0002), H-PE (P = 0.0132), and S-PE (P = 0.0197). Furthermore, the frequency of early preterm birth (< 34 GWs at delivery) was significantly lower among women with GH than among those with S-PE (P = 0.0025) and significantly lower among women with GP than among those with P-PE (P = 0.0109) and S-PE (P = 0.0035). The frequency of cesarean section was significantly lower among women with GP than among those with P-PE (P = 0.0137), H-PE (P = 0.0044), and S-PE (P = 0.0077) and significantly lower among women with GH than among those with H-PE (P = 0.0152) and S-PE (P = 0.0294). The frequencies of the HELLP syndrome (included partial type) and pulmonary edema were significantly lower among women with GH than among those with P-PE (P = 0.0014 and P = 0.0082, respectively), H-PE (P = 0.0116 and P = 0.0032, respectively), and S-PE (P = 0.0006 and P = 0.0005, respectively). The frequencies of abruptio placentae, eclampsia, and peripartum cardiomyopathy were similar among all groups. Furthermore, the frequencies of low weight for date among infants were similar among all groups.

### Trends of laboratory findings at the onset of gestational proteinuria or gestational hypertension, diagnosis of preeclampsia, and delivery

**Figure 1:**
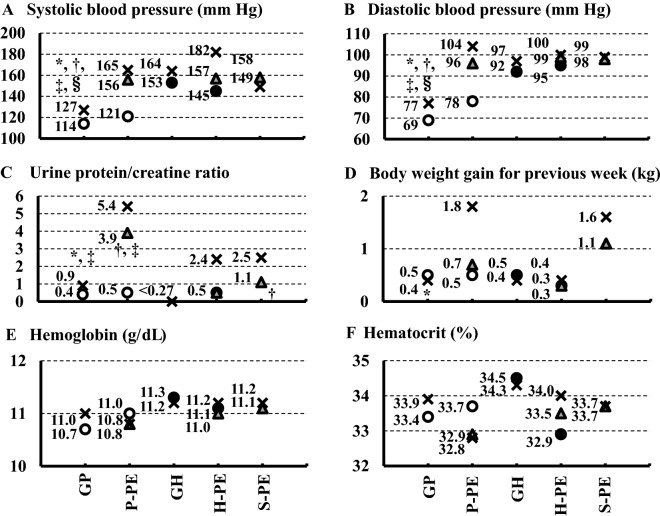
Trends of laboratory findings at onset of gestational proteinuria (GP) or gestational hypertension (GH), onset of preeclampsia, and delivery: systolic blood pressure (**A**), diastolic blood pressure (**B**), urine protein/creatine ratio (**C**), body weight gain for last 1 week (**D**), hemoglobin (**E**), and hematocrit (**F**). All data shown are median values. ○, at GP onset (appearance of proteinuria); ●, at GH onset (appearance of hypertension); ▲, at preeclampsia onset (hypertension plus proteinuria); ×, at delivery. *P < 0.05 versus proteinuria preceding preeclampsia (P-PE); ^†^P < 0.05 versus hypertension preceding preeclampsia (H-PE); ^‡^P < 0.05 versus simultaneous onset of proteinuria and hypertension before preeclampsia (S-PE); ^§^P < 0.05 versus GH.

**Figure 2:**
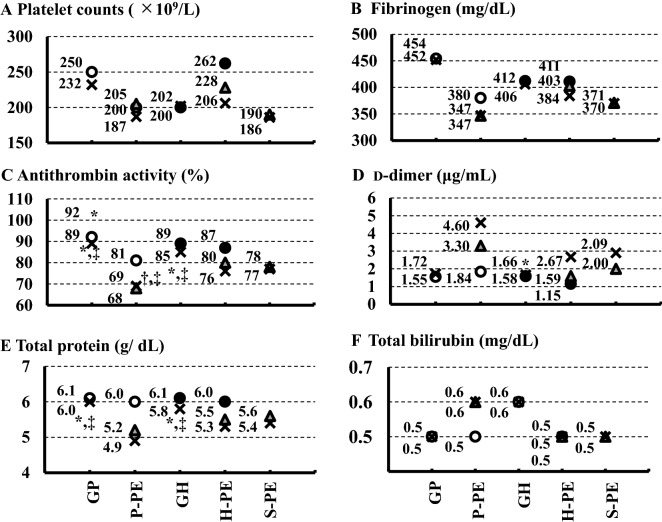
Trends of laboratory findings at onset of gestational proteinuria (GP) or gestational hypertension (GH), onset of preeclampsia, and delivery: platelet count (**A**), fibrinogen (**B**), antithrombin activity (**C**), d-dimer (**D**), total protein (**E**), and total bilirubin (**F**). All data shown are median values (or mean values). ○, at GP onset (appearance of proteinuria); ●, at GH onset (appearance of hypertension); ▲, at preeclampsia onset (hypertension plus proteinuria); ×, at delivery. *P < 0.05 versus proteinuria preceding preeclampsia (P-PE); ^†^P < 0.05 versus hypertension preceding preeclampsia (H-PE); ^‡^P < 0.05 versus simultaneous onset of proteinuria and hypertension before preeclampsia (S-PE); ^§^P < 0.05 versus GH.

**Figure 3:**
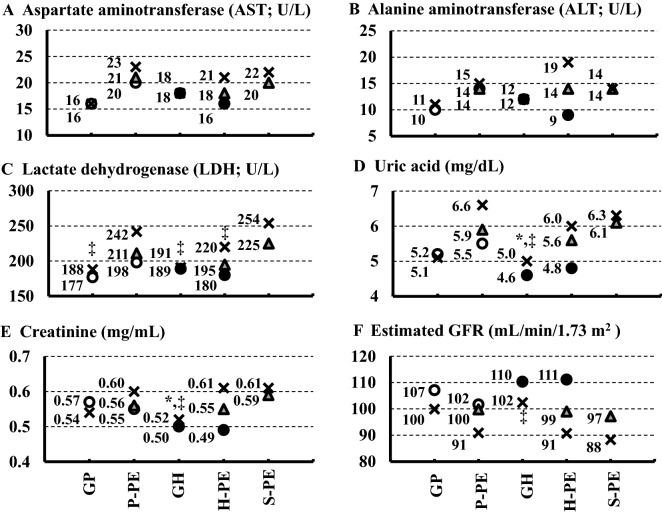
Trends of laboratory findings at onset of gestational proteinuria (GP) or gestational hypertension (GH), onset of preeclampsia, and delivery: aspartate aminotransferase (AST; **A**) alanine aminotransferase (ALT; **B**), lactate dehydrogenase (LDH; **C**), uric acid (**D**), creatinine (**E**), and estimated glomerular filtration rate (GFR; **F**). All data shown are median values. ○, at GP onset (appearance of proteinuria); ●, at GH onset (appearance of hypertension); ▲, at preeclampsia onset (hypertension plus proteinuria); ×, at delivery. *P < 0.05 versus proteinuria preceding preeclampsia (P-PE); ^†^P < 0.05 versus hypertension preceding preeclampsia (H-PE); ^‡^P < 0.05 versus simultaneous onset of proteinuria and hypertension before preeclampsia (S-PE); ^§^P < 0.05 versus GH.

Figures 1, 2 and 3 illustrate the differences in trends of laboratory findings at the onset of GP or GH, at the diagnosis of preeclampsia, and at delivery among women with GP, P-PE, GH, H-PE, and S-PE. Systolic (Fig. 1A) and diastolic blood pressures (Fig. 1B) at delivery of women with P-PE, S-PE, and GH were significantly higher than those of women with GP (all Ps < 0.0001). However, both systolic and diastolic blood pressures at the onset of proteinuria in women with P-PE were similar to those in women with GP, and those at the onset of hypertension in women with H-PE were similar to those in women with GH.

Urine protein/creatinine ratios (Fig. 1C) at delivery were significantly higher in women with P-PE than in those with GP (P = 0.0104), and those ratios at the diagnosis of preeclampsia were significantly higher in women with P-PE than in those with H-PE (P = 0.0168). However, urine protein/creatinine ratios at the time of the onset of proteinuria in women with P-PE were similar to those in women with GP.

Body weight gain for the week before delivery (Fig. 1D) was significantly higher in women with P-PE than in women with GH (P = 0.0492). Hemoglobin (Fig. 1E), hematocrit (Fig. 1F), platelet counts (Fig. 2A), and fibrinogen levels (Fig. 2B) did not differ among all groups.

Antithrombin activity (Fig. 2C) at the onset of proteinuria was significantly lower in women with P-PE than in those with GP (P = 0.0154) but similar at the onset of hypertension in women with H-PE and those with GH. Antithrombin activity at the diagnosis of preeclampsia was significantly lower in women with P-PE than in those with S-PE (P = 0.0115). Furthermore, antithrombin activity at delivery was significantly lower in women with P-PE and S-PE than in those with GP (P = 0.0006 and P = 0.0046, respectively) and significantly lower in women with P-PE and S-PE than in those with GH (P = 0.0003 and P = 0.0246, respectively).

D-dimer levels (Fig. 2D) at delivery were significantly higher in women with P-PE than those in women with GP and GH (P = 0.0232 and P = 0.0405, respectively). Total protein levels (Fig. 2E) at delivery were significantly lower in women with P-PE and S-PE than those in women with GP (P = 0.0010 and P = 0.0147, respectively) as well as in women with P-PE, H-PE, and S-PE than those in women with GH (P < 0.0001, P = 0.0303 and P < 0.0001, respectively). However, total protein levels in women with P-PE and S-PE at the onset of hypertension were significantly lower in women with GH (P < 0.0001 and P < 0.0001, respectively). Total bilirubin (Fig. 2F), aspartate aminotransferase (Fig. 3A), and alanine aminotransferase (Fig. 3B) levels did not differ among the five groups. Lactate dehydrogenase levels (Fig. 3C) were significantly higher at the diagnosis of preeclampsia in women with S-PE than those observed in women with H-PE (P = 0.0394) and significantly higher at delivery in women with S-PE than those in women with GP (P = 0.0194) and GH (P = 0.0086). Uric acid levels (Fig. 3D) at delivery were significantly higher in women with P-PE and S-PE than those in women with GH (P = 0.0015 and P < 0.0001, respectively). Similarly, creatinine levels (Fig. 3E) at delivery were significantly higher in women with P-PE and S-PE than those in women with GH (P = 0.0088 and P = 0.0005, respectively). The estimated glomerular filtration rate (Fig. 3F) at delivery was significantly higher in women with S-PE than that in women with GH (P = 0.0072).

### Cutoff value of laboratory findings to predict progression from presumptive gestation proteinuria to preeclampsia

Table 2 presents the optimal cutoff values of laboratory findings to predict progression from presumptive GP to preeclampsia (P-PE) based on the ROC curve. The univariate analysis provided the cutoff values for the following factors: the onset of proteinuria at 33.9 GWs (P = 0.0399), diastolic blood pressure of 62 mmHg (P = 0.0332), platelet count of 213 × 10^9^/L (P = 0.0229), fibrinogen level of 401 mg/dL (P = 0.0117), antithrombin activity of 82% (P = 0.0008), and lactate dehydrogenase level of 165 U/L (P = 0.0094). However, according to multivariate analysis, 33.9 GWs was the cutoff value for the onset of proteinuria. Furthermore, according to the multivariate logistic regression models, earlier onset of proteinuria (≤ 33.9 GWs) was the sole predictor for detecting progression from presumptive GP to preeclampsia (P-PE). The odds ratio (95% confidence interval) was 1.21 (1.21–1.48) (P = 0.0477).

**Table 2:** Relationship between laboratory cutoff values and progress from presumptive gestational proteinuria to preeclampsia.

Laboratory test	Cutoff value	Odds ratio	95% CI	AUC	Sensitivity	Specificity	PPV	NPV	P value	
									Univariate analysis	Multivariate analysis
Gestational weeks at the onset of proteinuria (weeks)	33.9	10.0	2.03–49.3	0.727	0.800	0.714	0.800	0.714	0.0399	0.0415
Systolic blood pressure (mmHg)	118	5.4	1.22–24.0	0.643	0.750	0.643	0.750	0.643	0.1235	
Diastolic blood pressure (mmHg)	62	30.0	1.50–602	0.675	1.000	0.429	0.714	1.000	0.0332	0.1300
Protein-to-creatinine ratio	0.70	2.44	0.51–11.6	0.545	0.400	0.786	0.727	0.478	0.4063	
Body weight gain per week (kg)	0.5	1.86	0.46–7.48	0.527	0.650	0.500	0.650	0.500	0.7486	
Hemoglobin (g/dL)	10.2	3.15	0.61–16.3	0.536	0.850	0.357	0.654	0.625	0.7071	
Hematocrit (%)	36.5	5.18	0.48–56.1	0.507	0.950	0.214	0.633	0.750	0.8874	
Platelet counts ($\times$ 10^9^/L)	213	11.1	1.92–64.5	0.743	0.650	0.857	0.867	0.632	0.0229	0.5870
Fibrinogen (mg/dL)	401	5.83	1.29–26.2	0.725	0.700	0.714	0.778	0.625	0.0117	0.7273
Antithrombin activity (%)	84	11.1	1.92–64.5	0.805	0.650	0.857	0.867	0.632	0.0008	0.1504
d-dimer (μg/mL)	1.74	2.00	0.50–8.00	0.529	0.600	0.571	0.667	0.500	0.8165	
Total protein (g/dL)	5.6	1.00	0.28–3.54	0.627	0.400	0.600	0.500	0.500	0.1554	
Total bilirubin (mg/dL)	0.7	5.57	0.59–52.7	0.620	0.300	0.929	0.857	0.482	0.2108	
Aspartate aminotransferase (U/L)	19	3.75	0.87–16.2	0.664	0.600	0.714	0.750	0.556	0.1303	
Alanine aminotransferase (U/L)	13	3.34	0.81–13.9	0.607	0.650	0.643	0.722	0.563	0.1125	
Lactate dehydrogenase (U/L)	165	30.0	1.50–602	0.727	1.000	0.429	0.714	1.000	0.0094	0.2530
Uric acid (mg/dL)	3.5	7.60	0.75–77.4	0.568	0.950	0.286	0.655	0.800	0.3971	
Creatinine (mg/dL)	0.42	10.9	0.50–238	0.554	1.000	0.214	0.645	1.000	0.4066	
Estimated glomerular filtration rate (mL/min/1.73 m^2^)	87.0	7.00	0.75–65.2	0.600	0.350	0.929	0.875	0.500	0.3322	

Laboratory findings were based on the receiver operating characteristic curve.

*CI* confidence interval, *AUC* area under the curve, *PPV* positive predictive value, *NPV* negative predictive value.

### Cutoff values of laboratory findings to predict progression from presumptive gestational hypertension to preeclampsia

Table 3 presents the optimal cutoff values of laboratory findings to predict the incidence of progression from presumptive GH to preeclampsia (H-PE) based on the ROC curve. According to univariate analysis, the cutoff values were 34.7 GWs at the onset of hypertension (P = 0.0031) and 0.0 kg as body weight gain during the week before delivery (P = 0.0497). However, according to multivariate analysis, 34.7 GWs was the cutoff value for the onset of hypertension. According to multivariate logistic regression models, earlier onset of hypertension (≤ 34.7 GWs) was the sole predictor for detecting the progression from presumptive GH to preeclampsia (H-PE). The odds ratio (95% confidence interval) was 1.21 (1.21–1.38) (P = 0.0044).

**Table 3:** Relationship between laboratory cutoff values and progress from presumptive gestational hypertension to preeclampsia.

Laboratory test	Cutoff value	Odds ratio	95% CI	AUC	Sensitivity	Specificity	PPV	NPV	P value	
									Univariate analysis	Multivariate analysis
Gestational weeks at the onset of proteinuria (weeks)	34.7	14.5	2.99–70.0	0.753	0.857	0.707	0.353	0.964	0.0031	0.0084
Systolic blood pressure (mmHg)	145	4.55	1.39–14.9	0.625	0.571	0.773	0.320	0.906	0.3065	
Diastolic blood pressure (mmHg)	100	3.53	0.43–29.0	0.523	0.929	0.213	0.181	0.941	0.3188	
Protein-to-creatinine ratio	< 0.27									
Body weight gain per week (kg)	0.0	4.00	1.22–13.2	0.657	0.500	0.800	0.318	0.896	0.0497	0.0543
Hemoglobin (g/dL)	11.9	2.82	0.59–13.6	0.549	0.857	0.320	0.191	0.923	0.6636	
Hematocrit (%)	36.0	3.18	0.66–15.3	0.591	0.857	0.347	0.197	0.929	0.2825	
Platelet counts ($\times$ 10^9^/L)	214	11.1	1.92–64.5	0.736	0.650	0.857	0.867	0.632	0.0357	
Fibrinogen (mg/dL)	375	2.18	0.56–8.51	0.527	0.786	0.373	0.190	0.903	0.5223	
Antithrombin activity (%)	100	6.43	0.36–115	0.520	1.000	0.187	0.187	1.000	0.7942	
d-dimer (μg/mL)	1.18	2.67	0.83–8.53	0.611	0.571	0.667	0.242	0.893	0.0998	0.3562
Total protein (g/dL)	6.4	5.4	0.67–43.8	0.598	0.929	0.293	0.197	0.957	0.4707	
Total bilirubin (mg/dL)	0.5	3.77	0.97–14.6	0.686	0.786	0.507	0.229	0.927	0.0272	0.3230
Aspartate aminotransferase (U/L)	17	3.36	0.97–11.7	0.664	0.714	0.573	0.238	0.915	0.0585	0.7110
Alanine aminotransferase (U/L)	9	3.01	0.94– 9.68	0.640	0.571	0.693	0.258	0.897	0.0585	0.1775
Lactate dehydrogenase (U/L)	205	3.00	0.62–14.5	0.585	0.857	0.333	0.194	0.926	0.3771	
Uric acid (mg/dL)	3.9	2.98	0.36–24.7	0.516	0.929	0.187	0.176	0.933	0.8385	
Creatinine (mg/dL)	0.45	2.06	0.64–6.69	0.556	0.429	0.733	0.231	0.873	0.8125	
Estimated glomerular filtration rate (mL/min/1.73 m^2^)	123.0	1.81	0.56–5.82	0.531	0.429	0.707	0.214	0.869	0.7445	

Laboratory findings were based on the receiver operating characteristic curve.

*CI* confidence interval, *AUC* area under the curve, *PPV* positive predictive value, *NPV* negative predictive value.

## Discussion

The results of this study emphasized the following four points: (1) Among women with preeclampsia, a large majority experienced S-PE, and the maternal and neonatal outcomes were similar among women with P-PE, H-PE, and S-PE; (2) preeclampsia was significantly more common in women with presumptive GP than in those with presumptive GH; (3) at the onset of proteinuria and at delivery, mean antithrombin activity was significantly lower in women with P-PE than that in women with GP; and (4) the predictor of progression from presumptive GP or GH to preeclampsia (P-PE or H-PE) was the onset of proteinuria or hypertension earlier in the pregnancy according to multivariate analysis based on the ROC curve.

According to the new Japanese criteria of “Hypertensive Disorders of Pregnancy (HDP)” revised in 2018^5^, preeclampsia is GH accompanied by at least one of the following new-onset conditions at or after 20 GWs; however, all symptoms normalized by 12 weeks postpartum. The primary new-onset condition is proteinuria. Further, other maternal organ dysfunctions, including the following, are considered new-onset conditions: (1) liver involvement without any underlying diseases (elevated transaminase levels, e.g., aspartate aminotransferase or alanine aminotransferase of > 40 IU/L, with or without severe persistent right upper quadrant or epigastric abdominal pain that cannot be diagnosed as other diseases and is treatment-resistant), (2) progressive kidney injury (creatinine levels of > 1.0 mg/dL, with other renal diseases being ruled out), (3) stroke and neurological complications (such as clonus, eclampsia, visual field disturbance, and severe headaches except for primary headache), (4) hematological complications (thrombocytopenia due to hypertensive disorders of pregnancy, resulting in a platelet count of < 150,000/μL, disseminated intravascular coagulation, and hemolysis). Another new-onset condition is uteroplacental dysfunction (involving fetal growth restriction, abnormal umbilical artery Doppler waveform, or stillbirth).

In the present study, of the 75 women with GH, 27 (36.0%) experienced other maternal organ dysfunctions and/or uteroplacental dysfunction. These 27 women were diagnosed with preeclampsia according to the new criteria of preeclampsia. Therefore, the new criteria of preeclampsia resulted in a 1.29-fold increase in the number of women diagnosed with preeclampsia. However, of the 94 women with preeclampsia (hypertension plus proteinuria), 52 (55.3%) experienced other maternal organ dysfunctions and/or uteroplacental dysfunction and 29 (30.9%) experienced uteroplacental dysfunction without any other maternal organ dysfunctions. Of all 121 women with preeclampsia according to the new criteria of preeclampsia, 42 (35.7%) women had preeclampsia as hypertension plus proteinuria alone was, however 94 (77.7%) women had preeclampsia as hypertension plus proteinuria and other maternal organ dysfunctions and/or uteroplacental dysfunction. Proteinuria might be the most important factor for the diagnosis of preeclampsia according to the new criteria of preeclampsia.

Bouzari et al. concluded that proteinuria in women with preeclampsia as diagnosed according to new criteria is associated with adverse outcomes of pregnancy, although it is not an adequate predictor^18^. However, in more recent studies, severe and massive proteinuria in women with preeclampsia as diagnosed according to new criteria were related to poor maternal and neonatal outcomes^19–21^.

In two previous studies, of women with new-onset GP, 24.7%^22^ and 33.7%^23^ developed preeclampsia; they tended to deliver earlier in the pregnancy, and their infants exhibited lower birth weights than those of women who remained normotensive. Therefore, patients with new-onset GP should be monitored for the development of preeclampsia and associated morbidity^24^.

Morikawa et al.^2^ reported that of 79 women who developed proteinuria (> 0.3 g/day) or hypertension, or both, at and after 20 GWs and gave birth between 2001 and 2005 in our institution, 37 (46.8%) had presumptive GP and 33 (41.8%) had presumptive GH. Presumptive GP progressed to P-PE significantly (3.4 times) more often (in 19 [51.4%] of 37 women) than did presumptive GH to H-PE (5 [15.2%] of 33 women; P = 0.002). These results were similar to those observed in the present study (progression to P-PE was 3.7 times higher and occurred in 58.8% of women with presumptive GP versus 15.7% of those with presumptive GH). Moreover, in the study by Morikawa et al.^2^, of 33 women with preeclampsia, 57.6% had P-PE, 15.2% had H-PE, and 27.3% had S-PE. The frequency of P-PE among all women with preeclampsia was significantly higher than that in our study (21.2%; P = 0.0003).

In the 2005 revision of the Japanese criteria for diagnosing preeclampsia, GP was not included among the hypertensive disorders of pregnancy. Therefore, presumptive GP might be paid little attention, and in some women, P-PE might be diagnosed as S-PE. The frequency of S-PE among all women with preeclampsia was significantly lower in Morikawa et al.’s study (27.3%) than in the present study (63.8%; P = 0.0005). In another previous report including 28 women with preeclampsia who gave birth between 2008 and 2013 in our institution^24^, Akaishi et al. observed that 10 women (35.7%) experienced P-PE. However, in that report, P-PE was considered a condition in which the duration of isolated GP was ≥ 3 days, and other preeclampsia (including H-PE and S-PE) was considered the condition in which the duration of isolated GP was ≤ 2 days. In a report of 111 women with preeclampsia who gave birth between 2008 and 2016 at another Japanese institution^25^, Nakamura et al. stated that 21 (18.9%) had P-PE, 48 (43.2%) had H-PE, and 42 (37.8%) had S-PE. S-PE was defined as onset of hypertension and proteinuria on the same day or within 2 days of one another after 20 weeks of pregnancy. In an observational multicenter study in Japan for 1 year (between 2014 and 2015), Yamada et al. reported that of 130 women with GP, 32 (24.6%) progressed to P-PE; these women accounted for 20.3% of all women with preeclampsia^26^. S-PE was defined as onset of both hypertension and proteinuria confirmed on the same day.

The previous studies raise some questions: do obstetricians monitor the protein/creatinine ratio of all pregnant women with GH every day or every other day? Do obstetricians monitor the laboratory findings of all pregnant women with isolated GP every day or every other day?

Eclampsia is one of severe maternal complications of preeclampsia, and the pathophysiological process is the same as that of preeclampsia. We speculated that proteinuria alone precedes eclampsia in some women without hypertension based on two previous reports from the United Kingdom. In one study, of 325 women with eclampsia in 1992, 186 (57%) experienced preeclampsia, 71 (22%) had GH, and 32 (9.8%) had GP alone^27^. In the other study, of 214 women with eclampsia between 2005 and 2006, 81 (38%) exhibited preeclampsia, 20 (9%) had GH, and 16 (7.5%) had GP alone^28^. In both reports, approximately 10% of the women tested exhibited proteinuria alone; thus, GP is a risk factor for preeclampsia as well as for eclampsia. The women exhibited proteinuria and hypertension at an antenatal visit within 1 week of their first convulsion. According to the recommendation by the National Institute for Health and Care Excellence of the United Kingdom, dipstick proteinuria testing was performed once or twice a week (with blood pressure measurement) in pregnant women with blood pressure values of 140/90–159/109 mmHg (hypertension) and daily in pregnant women who were admitted to a hospital with blood pressure value of ≥ 160/110 mmHg (severe hypertension)^29^. The National Institute for Health and Care Excellence of the United Kingdom^29^ and American College of Obstetricians and Gynecologists^1^ recommend that the blood examinations should be performed once a week; the International Society for the Study of Hypertension in Pregnancy^2^ recommends that blood examinations should be performed twice a week. Further, the Japan Society of Obstetrics and Genecology^30^ recommends that blood examinations should be performed once or twice a week. At Hokkaido University Hospital, the blood examinations were performed once or twice (or three times) a week for women with GH or GP.　Therefore, in the present study, preeclampsia was divided into the following three types: new-onset of hypertension/proteinuria at ≥ 20 GWs, additional new onset of other symptoms within 7 days (i.e., 1 week), or at ≥ 7 days (i.e., ≥ 1 week).

In a report from Italy, of 195 women with singleton pregnancies who had preeclampsia, compared with the 146 women (74.9%) who experienced H-PE, the 49 women (25.1%) who had P-PE experienced worse pregnancy-related and neonatal outcomes^31^. Furthermore, according to Akaishi et al., the pregnancy outcomes in women with P-PE were worse than those in women with H-PE or S-PE^24^. Nakamura et al. demonstrated that women with H-PE and S-PE have poorer perinatal prognoses than those with P-PE^25^.

Supplemental Fig. 2 shows GWs at GP or GH onset, preeclampsia onset, and delivery in the present study (Supplemental Fig. 2) and in previous reports (Supplemental Fig. 2B–D). The onset of proteinuria occurred significantly earlier in women with P-PE than in those with GP (P = 0.0276), and the onset of hypertension occurred significantly earlier in women with H-PE than in those with GH, both in our study (P = 0.0150; Supplemental Fig. 2A) and in Morikawa et al.’s study^2^ (Supplemental Fig. 2B). Preeclampsia was diagnosed, and delivery occurred significantly later among women with P-PE than among those with S-PE in Morikawa et al.’s study^2^ (Supplemental Fig. 2B) and among those with H-PE and S-PE in a report by Nakamura et al.^25^ (Supplemental Fig. 2C). According to Akaishi et al.^24^, preeclampsia was diagnosed significantly later in women with H-PE (34.4 weeks) and S-PE (36.0 weeks) than in those with P-PE (31.6 weeks), and the women with H-PE and S-PE delivered significantly later (35.5 and 36.7 weeks, respectively) compared with women with P-PE (32.5 weeks; not shown in Supplemental Fig. 2).

The abovementioned results differed from the present study findings. A study with a large sample size is necessary to evaluate the outcomes of women with P-PE. According to Yamada et al.^26^, preeclampsia was diagnosed and delivery occurred at similar times in women with P-PE, H-PE, and S-PE. Furthermore, the median GW at delivery was significantly later in women with H-PE than in those with GH; however, it was similar to that in women with GP (Supplemental Fig. 2D).

Akaishi et al. reported that the protein/creatinine ratios at birth were significantly inversely correlated with the antenatal lowest antithrombin activity and fibrinogen levels among the women with preeclampsia^24^. According to Morikawa et al.^2^, the antenatal lowest antithrombin activity was lower in women with P-PE than in those with H-PE, S-PE, and GP.

To the best of our knowledge, this is the first study to demonstrate the trends of laboratory findings at the onset of GP or GH, at the diagnosis of preeclampsia, and at delivery and to reveal the predictors (cutoff value of laboratory findings) of progression from presumptive GP or GH to preeclampsia. However, our findings would not be useful for predicting the progression of GP or GH to preeclampsia (hypertension plus proteinuria), except with regard to earlier onset of GP or GH.

Multicenter prospective randomized studies are necessary to determine the predictors (cutoff value of laboratory findings) of progression from presumptive GP or GH to preeclampsia. However, because the Japanese criteria for diagnosis of preeclampsia were revised in 2018, it would be challenging to perform such studies.

Our study has three major strengths. First, preeclampsia was diagnosed and treated in all the women in whom it was defined by hypertension plus proteinuria; thus, there was little bias (few confounding factors) regarding the criteria for diagnosing preeclampsia because maternal complications or placental dysfunction were excluded from these criteria. Second, all laboratory findings were obtained at the onset of proteinuria or hypertension, at the diagnosis of preeclampsia, and at delivery among women with GP, GH, or preeclampsia; therefore, the trends could be studied. Third, we were able to compare the frequencies of progression from presumptive GP or presumptive GH to preeclampsia as well as the frequencies of P-PE, H-PE, and S-PE among women with preeclampsia found in this study from 2009 to 2017 with those of Morikawa et al. obtained in an earlier study.

This study also has some limitations. First, it was performed in a single center, and the study population was small. Second, we did not measure the ratio of soluble fms-like tyrosine kinase 1 to placental growth factor; a previous study had shown that low ratios can be used to predict the short-term absence of preeclampsia in women in whom the syndrome was clinically suspected^32^. Third, our results were comparable only with those of other previous studies from Japan; similar previous studies from overseas were limited.

Earlier GW at onset of GP or GH was the best predictor of progression from presumptive GP to preeclampsia and from presumptive GH to preeclampsia. However, we could not determine the optimal cutoff values of general laboratory findings to predict progression from presumptive GP to preeclampsia and from presumptive GH to preeclampsia. Women with earlier onset of GP or GH should be considered to be at high risk for progression to subsequent preeclampsia.

## Methods

### Study design

This retrospective study was conducted at the Hokkaido University Hospital, Hokkaido, Japan. Patients in the cohort were 2904 pregnant women who had singleton pregnancies and delivered at 22 GWs or later between January 2009 and December 2017. This study was performed to clarify the cutoff levels of laboratory parameters as the predictors of progression from GP or GH to preeclampsia (Supplemental Fig. 1).

### Diagnosis of gestational proteinuria, gestational hypertension, and preeclampsia

GP, GH, and preeclampsia were diagnosed according to the classical criteria of the Japan Society of Obstetrics and Gynecology^1^. Hypertension was defined as systolic blood pressure of 140 mmHg or higher or diastolic blood pressure of 90 mmHg or higher. GH was defined as hypertension occurring at or after 20 GWs in the absence of significant proteinuria, defined as a protein/creatinine ratio higher than 0.27 on spot urine testing or higher than 0.3 g in a 24-h urine collection. GP was defined as significant proteinuria occurring at or after 20 GWs in the absence of hypertension. Preeclampsia was diagnosed in women who developed both hypertension and significant proteinuria at or after 20 GWs. Women with superimposed preeclampsia (chronic hypertension or proteinuria as an underlying disease before 20 GWs) were excluded from this study. A final diagnosis of preeclampsia required the absence of both hypertension and proteinuria at 12 weeks after delivery.

P-PE was defined as proteinuria preceding preeclampsia, in which proteinuria developed as the initial symptom at 20 GWs or later and hypertension then developed ≥ 7 days later. H-PE was defined as hypertension preceding preeclampsia, in which hypertension developed as the initial symptom at 20 GWs or later and proteinuria then developed ≥ 7 days later. S-PE was defined as the development of hypertension and proteinuria within 6 days of one another as the initial symptoms at 20 GWs or later.

### Management and termination of pregnancy among women with preeclampsia

All women with preeclampsia at Hokkaido University Hospital were admitted for treatment, and preeclampsia management (proteinuria measurements and blood examinations once or twice per week) was performed according to the recommendations of the guidelines of the Japan Society of Obstetrics and Gynecology^30^.

The pregnancy was terminated if maternal and fetal wellbeing deteriorated to the point at which the pregnancy could not be continued^1^ because of (1) severe hypertension (systolic blood pressure of ≥ 160 mmHg or diastolic blood pressure of ≥ 110 mmHg) that was non-responsive to antihypertensive agents; (2) emerging hypertension (systolic blood pressure of ≥ 180 mmHg or diastolic blood pressure of ≥ 120 mmHg); (3) HELLP syndrome (acute fatty liver of pregnancy, eclampsia, posterior reversible encephalopathy syndrome, pulmonary edema, peripartum cardiomyopathy, and abruptio placentae); or (4) poor fetal state documented on cardiotocography and biophysical profile score on ultrasonography. Furthermore, pregnancy termination was discussed for patients with severe proteinuria (protein/creatinine ratio of ≥ 5.0), severe kidney dysfunction (glomerular filtration rate of < 50 mL/min/1.73 m^2^), or abnormalities on maternal blood test results (e.g., low platelet count or antithrombin activity and high levels of aspartate aminotransferase, alanine aminotransferase, lactate dehydrogenase, and other enzymes).

### Inclusion and exclusion criteria

All pregnant women with GP, GH, or preeclampsia were included in this study. Pregnant women with preexisting chronic hypertension and superimposed preeclampsia were excluded.

### Laboratory findings

The maternal blood examinations and urine protein/creatinine ratio measurements were performed when GP or GH was diagnosed once or twice of times per week afterward. We analyzed laboratory findings (including hemoglobin and hematocrit levels; platelet count; antithrombin activity; fibrinogen, d-dimer, total protein, total bilirubin, aspartate aminotransferase, alanine aminotransferase, lactate dehydrogenase, uric acid, and creatinine levels; and estimated glomerular filtration rate). Furthermore, maternal systolic blood pressure, diastolic blood pressure, and body weight were measured at least once per day. The standard deviation of birth weight was calculated according to normative data for Japanese infants^33^.

### Statistical analyses

Data were calculated as median (25th percentile–75th percentile) or as frequencies. For statistical analyses, we used JMP Pro, version 14.0 (SAS Institute Inc., Cary, NC, USA). Kruskal–Wallis test or Mann–Whitney U test were used to compare the differences between the median values. Fisher’s exact test was used to compare categorical variables. ROC curves were used to assess the ability of parameters to differentiate the progress from GP to P-PE or the progress from GH to H-PE. A multiple logistic regression model was used in the multivariate analysis to compare the parameters that were significant in the univariate analysis (P < 0.1). P value of < 0.05 indicated statistical significance in all analyses.

### Ethical approval

This study was conducted after obtaining approval from the Institutional Review Board of Hokkaido University Hospital (Nos. 017-405 and 015-115). All participants provided written informed consent before the study. All methods were performed in accordance with Ethical Guidelines for Medical and Health Research Involving Human Subjects issued by Ministry of Education, Culture, Sports, Science and Technology and Ministry of Health, Labour and Welfare of Japan (http://www.lifescience.mext.go.jp/files/pdf/n1500_01.pdf).

## Supplementary Information

Supplementary Information 1.

Supplementary Information 2.

## Abbreviations

GH: Gestational hypertension
GP: Gestational proteinuria
GW: Gestational weeks
H-PE: Hypertension preceding preeclampsia
P-PE: Proteinuria preceding preeclampsia
S-PE: Simultaneous new onset of both hypertension and proteinuria before preeclampsia (“simultaneous preeclampsia”)
