# Supplementary material for: Earlier onset of proteinuria or hypertension is a predictor of progression from gestational hypertension or gestational proteinuria to preeclampsia
Source: Sci Rep. 2021 Jun 16;11:12708. doi: 10.1038/s41598-021-92189-w (PMC8209055; doi:10.1038/s41598-021-92189-w)
Supplement: Supplementary file 1 — Supplementary Information 1. [file 41598_2021_92189_MOESM1_ESM.pptx]

## Slide 1
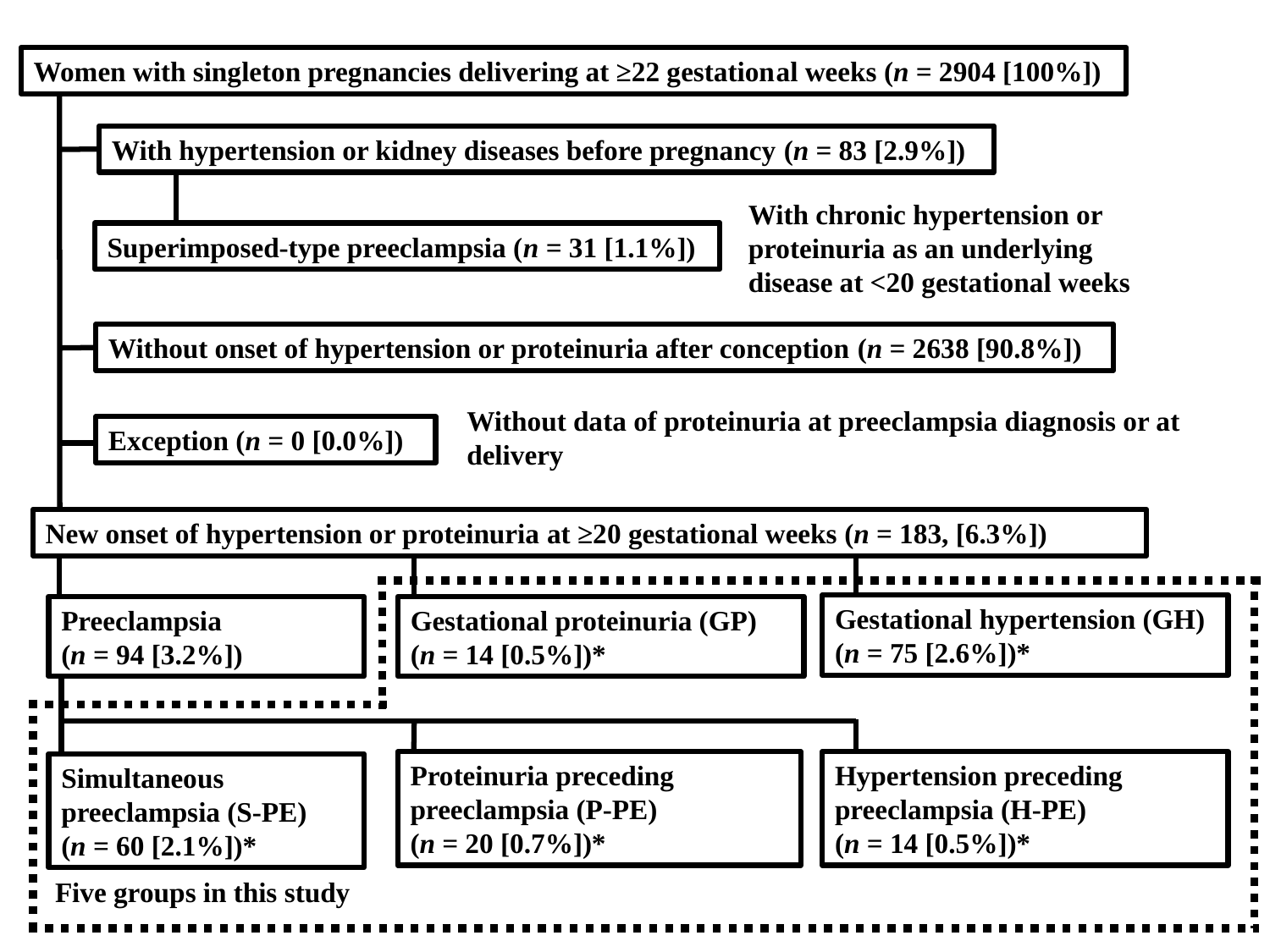

Women with singleton pregnancies delivering at ≥22 gestational weeks (n = 2904 [100%])
With hypertension or kidney diseases before pregnancy (n = 83 [2.9%])
With chronic hypertension or
proteinuria as an underlying
disease at <20 gestational weeks
Superimposed-type preeclampsia (n = 31 [1.1%])
Without onset of hypertension or proteinuria after conception (n = 2638 [90.8%])
Without data of proteinuria at preeclampsia diagnosis or at delivery
Exception (n = 0 [0.0%])
New onset of hypertension or proteinuria at ≥20 gestational weeks (n = 183, [6.3%])
Gestational hypertension (GH)
(n = 75 [2.6%])*
Preeclampsia
(n = 94 [3.2%])
Gestational proteinuria (GP)
(n = 14 [0.5%])*
Proteinuria preceding
preeclampsia (P-PE)
(n = 20 [0.7%])*
Hypertension preceding
preeclampsia (H-PE)
(n = 14 [0.5%])*
Simultaneous preeclampsia (S-PE)
(n = 60 [2.1%])*
Five groups in this study
Supplemental Figure 1. Study flowchart
*The subjects of this study were classified in five groups: those with gestational proteinuria (GP; n = 14); those with gestational hypertension (GH; n = 75); those with proteinuria preceding preeclampsia (P-PE; n = 20), in which proteinuria as the initial symptom occurred at 20 weeks of pregnancy or later and hypertension developed ≥7 days later; those with hypertension preceding eclampsia (H-PE; n = 14), in which hypertension as the initial symptom occurred at 20 weeks of pregnancy or later and proteinuria developed ≥7 days later; and those in whom both proteinuria and hypertension developed within 6 days of one another at 20 weeks of pregnancy or later and before preeclampsia (“simultaneous preeclampsia” [S-PE]; n = 60).
