# Supplementary material for: Earlier onset of proteinuria or hypertension is a predictor of progression from gestational hypertension or gestational proteinuria to preeclampsia
Source: Sci Rep. 2021 Jun 16;11:12708. doi: 10.1038/s41598-021-92189-w (PMC8209055; doi:10.1038/s41598-021-92189-w)
Supplement: Supplementary file 2 — Supplementary Information 2. [file 41598_2021_92189_MOESM2_ESM.pptx]

## Slide 1
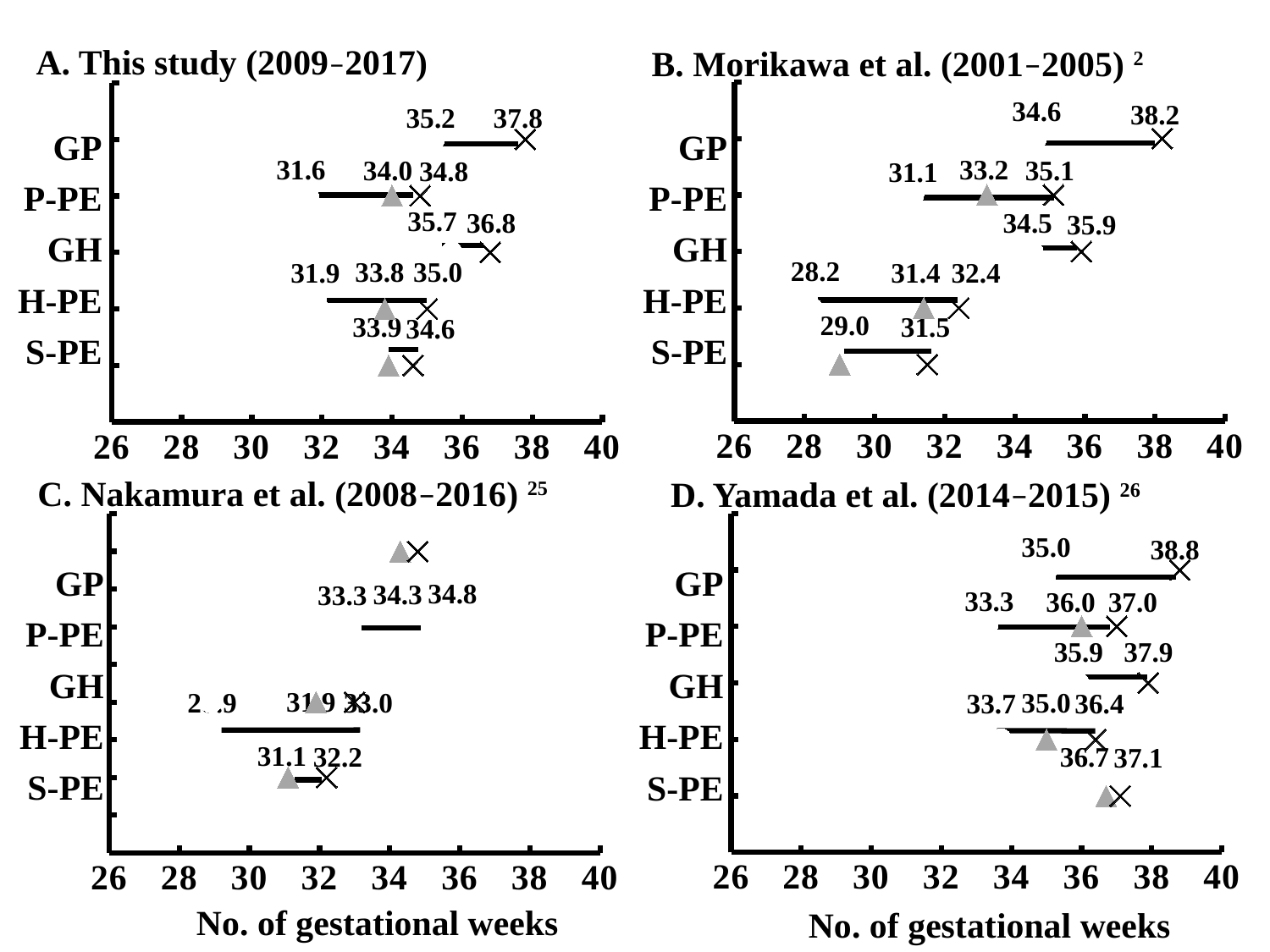

A. This study (2009–2017)
B. Morikawa et al. (2001–2005) 2
### Chart
| Category | |
|---|---|
### Chart
| Category | |
|---|---|34.6
38.2
35.2
37.8
GP
P-PE
GH
H-PE
S-PE
GP
P-PE
GH
H-PE
S-PE
33.2
31.6
34.0
35.1
34.8
31.1
35.7
36.8
34.5
35.9
28.2
33.8
35.0
31.9
32.4
31.4
29.0
33.9
31.5
34.6
C. Nakamura et al. (2008–2016) 25
D. Yamada et al. (2014–2015) 26
### Chart
| Category | |
|---|---|
### Chart
| Category | |
|---|---|35.0
38.8
GP
P-PE
GH
H-PE
S-PE
GP
P-PE
GH
H-PE
S-PE
34.8
34.3
33.3
33.3
37.0
36.0
35.9
37.9
31.9
28.9
33.0
35.0
33.7
36.4
31.1
32.2
36.7
37.1
No. of gestational weeks
No. of gestational weeks
Supplemental Figure 2. Gestational weeks at onset of gestational proteinuria (GP) or gestational hypertension (GH), onset of preeclampsia, and delivery. A. This study (2009–2017). B. Previous study at the same institution (2001–2005) 2. C. Study at another institution in Japan (2008–2016) 25. D. Multicenter observation study in Japan (2014–2015) 26.
All of data shown are means. ○, at GP onset (appearance of proteinuria); ●, at GH onset (appearance of hypertension); ▲, at preeclampsia onset (hypertension plus proteinuria); ×, at delivery. *P < 0.05 versus P-PE (proteinuria preceding preeclampsia), in which proteinuria as the initial symptom occurred at 20 weeks of pregnancy or later and hypertension developed ≥7 days later. †P < 0.05 versus H-PE (hypertension preceding eclampsia, in which hypertension as the initial symptom occurred at 20 weeks of pregnancy or later and proteinuria developed ≥7 days later). ‡P < 0.05 versus S-PE (both proteinuria and hypertension developed within 6 days of one another at 20 weeks of pregnancy or later and before preeclampsia [“simultaneous preeclampsia”]). §P < 0.05 versus GH.
